# Empirical Comparisons of Different Statistical Models To Identify and Validate Kernel Row Number-Associated Variants from Structured Multi-parent Mapping Populations of Maize

**DOI:** 10.1534/g3.118.200636

**Published:** 2018-09-25

**Authors:** Jinliang Yang, Cheng-Ting “Eddy” Yeh, Raghuprakash Kastoori Ramamurthy, Xinshuai Qi, Rohan L. Fernando, Jack C. M. Dekkers, Dorian J. Garrick, Dan Nettleton, Patrick S. Schnable

**Affiliations:** * Department of Agronomy; § Department of Animal Science and Center for Integrated Animal Genomics; ** Department of Statistics, Iowa State University, Ames, IA 50011; † Department of Agronomy and Horticulture, University of Nebraska-Lincoln, Lincoln, NE 68583; ‡ Department of Ecology and Evolutionary Biology, University of Arizona, Tucson, AZ

**Keywords:** GWAS KRN maize Bayesian, Multiparent Advanced Generation Inter-Cross (MAGIC), multiparental populations, MPP

## Abstract

Advances in next generation sequencing technologies and statistical approaches enable genome-wide dissection of phenotypic traits via genome-wide association studies (GWAS). Although multiple statistical approaches for conducting GWAS are available, the power and cross-validation rates of many approaches have been mostly tested using simulated data. Empirical comparisons of single variant (SV) and multi-variant (MV) GWAS approaches have not been conducted to test if a single approach or a combination of SV and MV is effective, through identification and cross-validation of trait-associated loci. In this study, kernel row number (KRN) data were collected from a set of 6,230 entries derived from the Nested Association Mapping (NAM) population and related populations. Three different types of GWAS analyses were performed: 1) single-variant (SV), 2) stepwise regression (STR) and 3) a Bayesian-based multi-variant (BMV) model. Using SV, STR, and BMV models, 257, 300, and 442 KRN-associated variants (KAVs) were identified in the initial GWAS analyses. Of these, 231 KAVs were subjected to genetic validation using three unrelated populations that were not included in the initial GWAS. Genetic validation results suggest that the three GWAS approaches are complementary. Interestingly, KAVs in low recombination regions were more likely to exhibit associations in independent populations than KAVs in recombinationally active regions, probably as a consequence of linkage disequilibrium. The KAVs identified in this study have the potential to enhance our understanding of the genetic basis of ear development.

Following the adoption of genome wide association studies (GWAS) (Klein *et al.* 2005), ∼2,000 loci have been identified as being statistically associated with human disease and other quantitative traits (Visscher *et al.* 2012). Using GWAS approaches, hundreds of loci associated with traits have been identified in crops including maize (Brown *et al.* 2011; Tian *et al.* 2011; Leiboff *et al.* 2015), rice (Huang *et al.* 2010), sorghum (Morris *et al.* 2013) and barley (Cockram *et al.* 2010), and in non-crop models such as *Arabidopsis* (Atwell *et al.* 2010; Meijón *et al.* 2014).

There are multiple statistical models for conducting GWAS, including both single-variant (SV) and multi-variant (MV) models. SV analysis compares the phenotypic distributions of alternative genotypes at each polymorphic site independently. They can be conducted without correction for population structure or with correction using techniques such as genomic control (Devlin and Roeder 1999) or principal component analysis (Price *et al.* 2006). More recently, genetic relatedness among individuals can be accounted using a kinship matrix in mixed linear models (Yu *et al.* 2006). Although SV analysis is often used in published literature, it has a number of inherent limitations, such as not being able to distinguish among the contributions of closely linked loci (Yang *et al.* 2012). Sometimes SV analysis overcorrects for the genomic inflation of the test statistics caused by genetic structure because covariates identified via population structure analysis can be associated with causal loci (Yang *et al.* 2011). In comparison, MV models have already been demonstrated to be superior in classical linkage analyses, where for example, composite interval mapping outperforms simple interval mapping (Zeng 1993). MV models can explicitly account for large-effect loci and estimate the effects of multiple loci simultaneously. It has been suggested that the power of GWAS may be improved by conditioning on major-effect QTL (Kang *et al.* 2010). One challenge to using MV models, however, is the substantial computational burden associated with analyzing a large number of polymorphic sites. As a partial solution, stepwise regression, which selects markers based on forward inclusion and backward elimination, has been proposed (Segura *et al.* 2012). As an alternative to stepwise regression, multi-variant Bayesian models that were initially developed for genomic prediction, by simultaneously fitting all genotyped loci across the genome (Meuwissen *et al.* 2001), have been used for GWAS (Fan *et al.* 2011; Habier *et al.* 2011). These Bayesian-based approaches fit hierarchical models that allow the effects of many loci to shrink toward zero. An empirical comparison of the above models is necessary to investigate their relative advantages in dissecting the genetic architecture of target traits (either individually or in combination).

Many models for conducting GWAS have been compared using simulated data (Galesloot *et al.* 2014). Although such studies can provide insight, they suffer from the limitation that simulated data do not necessarily reflect all characteristics of real data because some characteristics of empirical data may be unknown to the simulator. Hence, comparisons based on empirical data are complementary to those based on simulated data. Other than studies that compared different versions of mixed linear model approaches (Stich *et al.* 2008), to our knowledge there are no published reports that compare results generated from empirical data analyzed using single-variant (SV), stepwise regression (STR) and Bayesian-based multi-variant (BMV) models for GWAS. In this study, our objective is to compare the effectiveness and/or complementarity of different GWAS (SV, STR, and BMV) models using a single empirical data set. Our second objective is to assess the degree to which our findings from different GWAS models would support other independent empirical data sets.

We perform our initial GWAS analyses on four populations (described in **Material and Methods**) related to the nested association mapping (NAM) population, which combines the strengths of historical recombination events across a broad base of genetic diversity and recombination events that arise following experimental crosses (Yu *et al.* 2008). Rather than studying diversity panels often featured in GWAS, we consider multi-parent mapping populations to take advantage of the statistical power of QTL mapping and the gene-level resolution of association mapping.

We study the kernel row number (KRN) phenotype, which is both a component of yield and a model trait for genetic studies in maize (Hallauer *et al.* 2010). KRN is highly heritable and a polygenic trait (Liu *et al.* 2015a,b) that exhibits little variation in response to environment (Lu *et al.* 2011). In addition, KRN is easily scored as an integer, and this scoring can be conducted after completion of the busy pollination season. We collected new KRN data and downloaded previously published KRN data (Brown *et al.* 2011).

Collectively, the three GWAS models identified 988 unique KRN-associated variants (KAVs), 231 of which were subjected to genetic validation tests using three unrelated populations that were not included in the initial GWAS. Approximately 60% of the validated KAVs were identified by only one of the three approaches.

## Materials and Methods

### KRN phenotyping for initial GWAS populations

KRN phenotypes were collected from four related populations: 1) the nested association mapping (NAM, N = 4,699) population which was composed of 25 recombinant inbred line (RIL) subpopulations (Yu *et al.* 2008), plus RILs of intermated B73 and Mo17 (IBM, N = 325) (Lee *et al.* 2002), 2) a subset of the RILs that were backcrossed to the inbred line B73 (B73 x RILs, N = 692 BC1 lines), 3) a subset of the RILs that were backcrossed to the inbred line Mo17 (Mo17 x RILs, N = 289 BC1 lines) and 4) a partial diallel created from the 26 NAM founders and Mo17 (N = 225 F1 hybrids). Because reciprocal crosses were not considered and some of the crosses were not successful, the diallel population was both partial and incomplete (225/351 = 64%).

During the years 2008-2011, NAM RIL populations were sequentially planted in Ames, IA (summer season) and in Molokai, HI (winter season), such that only a fraction of the full set of RILs were grown in a given environment. For each NAM RIL, five plants were grown within each row. KRN data were collected from the mature ears. In addition, we obtained KRN data from eight trials for NAM RILs from a previous study (Brown *et al.* 2011). The Brown *et al.* (2011) data set is eight times larger than ours. We obtained final phenotypic values by fitting a mixed linear model with fixed effects for entries and random effects for trials. Phenotypic density distributions in this study were estimated and plotted using R with default smoothing parameters.

During the summer of 2011, the B73 x RILs, Mo17 x RILs, and the diallel populations (GWAS populations 2-4) were planted in Ames, IA in 12-plant rows. The B73 x RILs and Mo17x RILs were planted with two replications, and the diallel population was planted with three replications. For each replication, KRN data were collected from three mature ears. The obtained KRN phenotypic data were analyzed using a mixed linear model, where genotype was fitted as a fixed factor and replication was fitted as a random factor.

### KRN phenotyping for subsequent validation populations

Elite maize inbred lines, extreme KRN USDA accessions, and lines obtained from Iowa long ear synthetic (BSLE) population were used for genetic validation of KAVs identified through GWAS. A total of 220 elite inbred lines, commercial lines that had formerly been subject to IP (Intellectual Property) protection via the plant variety protection act, were obtained from the USDA Plant Introduction (PI) Station in Ames, IA (http://www.ars.usda.gov/main/site_main.htm?modecode=36-25-12-00). During year 2011, these accessions were planted in three replications and observed for KRN phenotypes. About 7,000 of the maize accessions have been phenotyped for the KRN trait in the database of USDA PI Station. We selected the 225 accessions with the largest KRN values, the 208 accessions with the smallest KRN values, and 173 random accessions to serve as the second genetic validation population. Empirical KRN phenotypes were obtained from our previously published replicated field experiment (Yang *et al.* 2015). Because of the genetic heterogeneity of the accessions, up to 12 random seeds were germinated and pooled together for each accession for DNA isolation. The BSLE population was the product of a long-term selection project conducted to divergently select long and short ears from a single founder population (Hallauer *et al.* 2004). Parental lines (N = 10/12) of BSLE and bulked seeds from cycle 0 (C0), cycle 30 short ear (C30 SE) and cycle 30 long ear (C30 LE) were obtained from Arnel Hallauer as our third validation population.

### Genomic variant processing

A set of 6.2 million genic variants (SNPs and small InDels) was identified via analysis of RNA-seq data from five tissues (shoot apical meristem, ear, tassel, shoot and root) on 26 NAM founder lines and Mo17 (Lin *et al.* 2017). Another two sets of genomic variants generated from the maize HapMap project (Bukowski *et al.* 2015) were extracted from the Panzea database (https://www.panzea.org). These three sets of variants were merged using the consensus mode of PLINK (Purcell *et al.* 2007). The merged variants were further filtered by discarding variants with a call rate of < 0.4 across entries. We further filtered variants using a minor allele frequency (MAF) cutoff of < 0.1 to exclude minor SNPs only present in fewer than five non-B73 parents. The finalized set consists of 12,966,279 genomic variants on NAM founders, which were used for imputation or projection onto the four related GWAS populations.

Imputation of genotypes was performed as described below. NAM RILs had been directly genotyped using genotyping-by-sequencing (GBS) technology (Elshire *et al.* 2011). We obtained the GBS data from the Panzea database (https://www.panzea.org). Based on the GBS data and the known pedigrees, the ∼13 million variants discovered in the NAM founders were imputed onto NAM RILs using a python script (https://github.com/yangjl/zmSNPtools) as previously described (Yu *et al.* 2008). Because the B73xRIL, Mo17xRIL and partial diallel populations were composed of pairs of known haplotypes, their genotypes were directly projected from their parents using the above python script.

### Association variants thinning

Because progeny of bi-parental crosses comprised much of the initial GWAS population, the strong linkage of genetic variants violated the assumption of independence needed to determine statistical-based thresholds. Therefore, a variant thinning procedure was developed to select the most significant variants, and to avoid concentration of selected variants in certain regions. For variants located in the 28 QTL intervals from the joint QTL analysis and their 1-Mb flanking regions, the top 10 most significant variants were selected. For variants located in other regions, significant variants were determined following the arbitrary thresholds: $-log_{10}\left(P\right)>20$ for the SV model, posterior model frequency (MF) > 0.02 for the BMV model and an inclusion *P* value < 0.05 for the stepwise regression. These significant variants were clustered as groups if none of their pair-wise physical distances exceeded 10-Mb. From these clustered groups, no more than 10 most significant variants were selected.

### Amplicon sequencing for validation of KAVs

Amplicon sequencing assays were designed for 140/231 (61%) KAVs. A total of 1,102 DNA samples from elite inbred lines (N = 208), extreme KRN lines from the USDA germplasm collection (N = 606) and individuals from the Iowa Long Ear Synthetic (BSLE, N = 288) were individually genotyped, by sequencing all multiplexed amplicons from all 1,102 samples in one HiSeq2000 lane. Informative variants, defined as those which were successfully genotyped, were polymorphic, and had a call rate of > 0.4 and a MAF > 0.05 were used for genetic validation.

#### Statistical Analysis:

##### Joint QTL analysis

Joint linkage analysis was performed on NAM and IBM RILs using their corresponding genetic maps. A two-step composite interval mapping (CIM) (Zeng 1993) method was employed using a suite of programs within QTL cartographer (Silva Lda *et al.* 2012). First, an automatic forward stepwise regression procedure was used to sequentially test all SNP markers; the most significant marker (inclusion threshold = 0.05) was kept after each iteration. This procedure was repeated until no SNP met the inclusion threshold. In the second step, linkage analyses were conducted at 1-Mb intervals along the chromosome treating previously selected SNPs (other than those within the 1-Mb interval under analysis) as co-variates. A significance threshold was determined by conducting 1,000 permutations and QTL confidence intervals were defined using a 1.5-LOD drop from QTL peak (Lander and Botstein 1989).

To account for documented stratification effects, the statistical model included fixed effects for population and subpopulation for all three GWAS models described below.

##### Single-variant (SV) GWAS model

Additional fixed effects were fitted in the model to control for effects of QTL on other chromosomes, while all variants on a single chromosome were scanned, resulting in the following model 1 for the *k*th variant:

$$Y_{l}=u_{k}+\sum_{i=1}^{4}a_{ik}P_{il}+\sum_{j=1}^{26}b_{jk}S_{jl}+\sum_{m\in Ch\left(k*\right)}c_{km}Q_{ml}+d_{k}VAR_{kl}+e_{kl}$$ (1)

where $Y_{l}$ is the adjusted KRN phenotypic value for line *l* from the mixed linear model analysis; $u_{k}$ is an intercept parameter; $P_{il}$ is 1 if line *l* is of GWAS population *i* and is 0 otherwise, and $a_{ik}$ is the effect of the *i*th population in the model for variant *k*; $S_{jl}$ is 1 if line *l* is from subpopulation *j* and 0 otherwise, $b_{jk}$ is the effect of subpopulation *j* in the model for variant *k*; $Q_{ml}$ indicates the line *l* genotype of the *m*th QTL detected by the joint linkage analyses, $c_{km}$ is the effect of the *m*th QTL in the model for variant *k*, Ch(k∗) is the set of QTL detected by the joint linkage analysis that are located on chromosomes other than the chromosome of variant *k*; $VAR_{kl}$ indicates the genotype of the *k*th variant in line *l*, $d_{k}$ is the effect of the *k*th variant; and $e_{kl}$ is an error term. This SV model 1 was implemented using SNPTEST v2.3.0 (Marchini and Howie 2010).

##### Stepwise regression (STR) GWAS model

In the stepwise regression test, population and subpopulation effects were fitted first as fixed effects, and then markers were added in a stepwise manner. For each marker, $R^{2}$ was calculated as the proportion of sums of squares after the fixed effects. We employed the STR method that was implemented in GenSel v4.1 (Habier *et al.* 2011) with the option of “StepWise”. We used the following three options to control the STR model, 1) inputMaxRsquared (default 0.8), inputMaxMarkers (300) and alphaValue (default 0.05).

##### Bayesian-based multi-variant (BMV) GWAS model

A Bayesian-based MV model was constructed using the “BayesC” option of GenSel v4.1 (Habier *et al.* 2011). This model differs from the SV model in that it estimates the effects of all variants simultaneously rather than testing them one-at-a-time. The effects of the variants were fitted as random effects. The following mixed model was used.

$$Y_{l}=u+\sum_{i=1}^{4}a_{i}P_{il}+\sum_{j=1}^{26}b_{j}S_{jl}+\sum_{k}^{∼13M}c_{k}VAR_{kl}+e_{l}$$ (2)

where $VAR_{kl}$ indicates the genotype of the *k*th variant in line *l* and $c_{k}$ is the effect of the *k*th variant; other terms in the model are as described in the SV model 1 except that neither the *u*, $a_{i},$ or $b_{j}$ parameters nor the $e_{l}$ error terms are specific to the *k*th variant in the BMV model 2.

We trained the BMV model using a two-step procedure. In the first step, we ran 1,000 iterations with 100 burn in of MCMC simulation using default priors, *i.e.*, genetic variance (genVariance = 1) and residual variance (resVariance = 1). In the 2^nd^ step, we replaced the priors using the posteriors obtained from step 1 and ran a longer chain of simulations (chainLength = 41,000 and burnIn = 1,000).

### Data Availability

File S1 contains phenotypic data for the GWAS populations. File S2 contains the KAVs identified using the three GWAS models. File S3 contains genotypes of KAVs for the three validation populations. File S4 contains the KRN phenotypic data for the validation populations. In the table, “Internal_id” could be used as the identifier to match genotypic data. File S5 contains the genetic validation results. Stars indicate SNPs that were consistently associated with KRN in initial GWAS, and in at least one of the validation populations. All the supplementary files have been uploaded to Figshare (10.6084/m9.figshare.6902144). R code for the analyses is available in the public GitHub repository (https://github.com/yangjl/KRN-GWAS). Supplemental material available at Figshare: 10.6084/m9.figshare.6902144.

## Results

### KRN Phenotype in GWAS and Validation Populations

**Figure 1:**
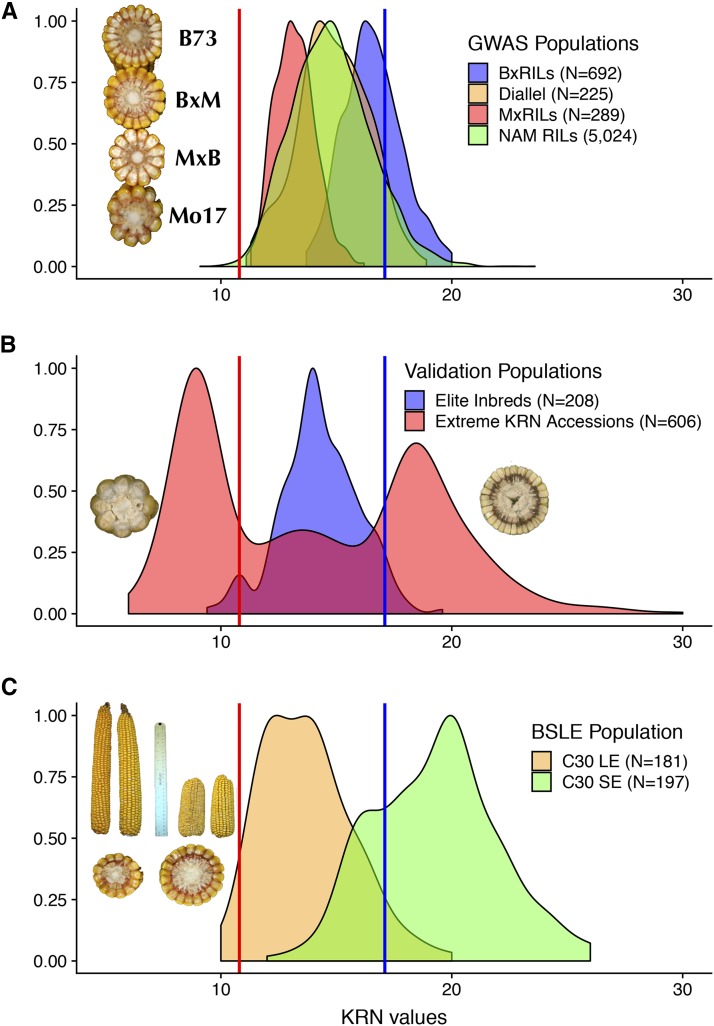
Phenotypic distributions of the KRN trait in GWAS and validation populations. (A) Density plots of the four GWAS populations. Embedded picture shows the typical KRN counts for B73, B73xMo17 (BxM), Mo17xB73 (MxB) and Mo17 lines. (B) Density plots of two validation populations, elite inbred lines and extreme KRN accessions. Embedded pictures shows examples of an extreme low KRN accession and an extreme high KRN accession. (C) Density plots of cycle 30 long ear (C30 LE) and cycle 30 short ear (C30 SE) in the BSLE population. Embedded pictures indicate the ear length and KRN variation after 30 generations of selection. Blue and red dashed lines indicate the mean KRN values of B73 (KRN = 17.1) and Mo17 (KRN = 10.8).

We collected KRN phenotypic data from 6,230 entries within four related GWAS populations grown at two locations over four years (see Materials and Methods). Best linear unbiased estimators (BLUE) of the KRN phenotype were calculated for each of the 6,230 entries in the four GWAS populations (File S1). In this combined analysis, KRN phenotypic values ranged from 9.1 to 23.6, with a mean of 14.9 rows, whereas the B73 inbred had an above average KRN phenotype of 17.1 rows. Density plots of the four GWAS populations exhibited the expected bell-shaped distributions (Figure 1A).

We also collected KRN data from three unrelated populations for subsequent validation purpose. The three populations consist of elite inbred lines that expired from US plant variety protection (Nelson *et al.* 2008), extreme KRN accessions from USDA germplasm database, and a long term selection population — Iowa long ear synthetic (BSLE). The elite lines have KRN that are less extreme and less variable than the NAM lines (Figure 1B). This fits with our understanding of breeder practices; they do not select for high KRN phenotypes. The USDA Plant Introduction station maintains a large collection of maize germplasm. The KRN phenotypes in this population are extreme, ranging from an average of 13-30 rows in the high KRN pool to 6-12 rows in the low KRN pool according to data obtained from a replicated field trial (Figure 1B) (Yang *et al.* 2015). The BSLE population had been subjected to 30 generations of divergent selection for long ears (LE) and short ears (SE) (Hallauer *et al.* 2010). During selection, KRN exhibited a negatively correlated response to ear length (r=−0.6, Pearson’s correlation test *P* value <0.05), *i.e.*, longer and shorter ears had smaller and larger KRN trait values, respectively (Figure 1C). The parental lines and bulked seeds from cycle 0 (C0), cycle 30 long ear (C30 LE) and cycle 30 short ear (C30 SE) served as our third genetic validation population.

### Identification of KAVs With different statistical models using GWAS populations

**Figure 2:**
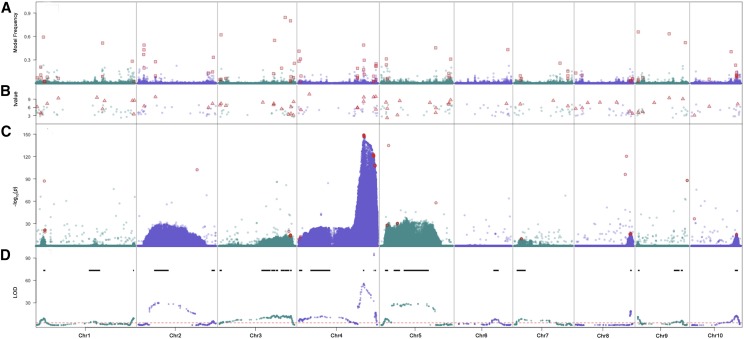
Stacked plots of GWAS and QTL results. From upper to lower panels are results from the Bayesian-based multi-variant (A) stepwise regression (B) and single variant(C) models for GWAS and the joint QTL mapping result (D). The red dashed line in the QTL plot indicates the 1,000 permutation threshold and black lines show the QTL confidence intervals. Red squares in panel (A), triangles in panel (B) and circles in panel (C) indicate the kernel row number associated variants selected for further genetic validation.

In each GWAS model, population and subpopulation were included as fixed effects to account for inherent structure in the 6,230 entries included in the GWAS. First, a SV model (Manolio 2010) was used to scan the ∼13M variants one-by-one using QTL detected in the joint analysis as covariates. The linear mixed model with genetic relationship matrix was not used here because the four initial GWAS populations are NAM and NAM related multi-parent mapping populations. Using an arbitrary cutoff of $-log_{10}\left(P\right)>20,$ this approach identified linked clusters of variants (Figure 2C), most of which were located within the 28 QTL intervals that had been identified by the joint QTL analysis (Figure 2D). To diminish the over-representation of certain regions by significant variants, a binning (bin size = 100-kb) procedure was used that resulted in the identification of 257 KAVs, which in combination accounted for 51% of the KRN variation.

Second, in an attempt to improve mapping resolution, a STR approach was used, which resulted in identification of 300 KAVs representing 296 100-kb bins (Figure 2B). In combination, these variants accounted for 78% of phenotypic variation.

Third, a BMV model (Habier *et al.* 2011) was used to estimate effects of all ∼13M variants simultaneously. After applying the variant thinning procedure and cutoffs described in Materials and Methods, a set of 442 variants representing 343 100-kb bins, which together accounted for 74% of the phenotypic variation, was identified (Figure 2A). Most promisingly, this model identified smaller chromosomal intervals than the SV model.

### Comparison of KAVs identified by three GWAS Models

In combination, the three GWAS models identified 764 100-kb bins (File S2), each of which contained one or more significant variants. Encouragingly, among these 764 bins, 66 (containing 169 variants) were detected by at least two models. Only one of these bins was detected by all three GWAS models. That bin (chr4:229.0-Mb) overlaps the most significant QTL peak detected in the joint QTL study. To estimate an upper bound for cross-validation rate for each approach and to determine whether the KAVs that were detected by more than one approach are more reliable, a set of 231 KAVs was selected for genetic validation. This set of KAVs included the 169 variants in the 66 bins detected by at least two approaches and 62 of the most significant one or two variants selected from 20 bins that had only been detected by one approach (approach-specific variants). Hence, in total 231 KAVs from a total of 126 bins (66 + 20 × 3) were selected for genetic validation.

Collectively, the 231 selected KAVs explained 64% of phenotypic variation in the initial GWAS population by fitting these KAVs simultaneously using an additive model (*i.e.*, narrow-sense heritability $h^{2}\approx 64\text{\%}$) . Individually, most of the KAVs (83%, 192/231) explained less than 5% of the phenotypic variation, but 17% (39/231) of the KAVs individually accounted between 5–10% of phenotypic variation.

Note that a causal variant might be represented by multiple selected KAVs. The B73 variant-types were alleles for higher KRN for nearly three-quarters (73%, 168/231) of these KAVs, consistent with the high KRN value of the B73 genotype. Consistent with our previous study (Li *et al.* 2012), KAVs are substantially enriched for variants located within genes or within 5-kb upstream of genes (2.0-fold change, Chi-square *P* value < 0.01) and enriched in variants discovered from the RNA-seq data (1.ninefold change, Chi-square *P* value < 0.01) relative to the ∼13M variants used for GWAS.

### Genetic validation of KAVs using three unrelated populations

To distinguish true positive association signals from potentially false positive associations, three previously described genetic validation populations that are unrelated to the GWAS populations and to each other were genotyped at the KAVs (genotype data are provided in File S3 and S4).

To control for population structure in the elite inbred lines, a set of SNPs that had previously been used to genotype a subset (N = 91) of these lines was obtained (Nelson *et al.* 2008). We fitted a mixed linear model to estimate the fixed effects of KAVs. In the model, we included random effects for lines. These random effects were assumed to be correlated according to a kinship matrix calculated from the genome-wide SNPs (Nelson *et al.* 2008). Using this approach, 22/70 (31%) of the informative KAVs could be genetically validated in the set of 91 elite inbreds with an FDR < 0.05. Because the elite inbreds are not closely related to the GWAS populations, it is unlikely that uncontrolled population structure could yield false-positive validation assays for KAVs derived from the GWAS populations. Hence, we also conducted a naive analysis using the entire set of elite inbreds (N = 209) without controlling for population structure. In this analysis, 33/70 (47%) of the KAVs, which included all of the 22 KAVs discussed above, could be validated.

Because extreme KRN accessions were maintained via random pollination within accessions, individual accessions are both heterogeneous and heterozygous. We therefore genotyped pools of DNA extracted from up to 12 plants per accession. A model fitted to the estimated allele frequencies was used to test the hypothesis that alleles for higher KRN have higher frequencies in the high KRN pools than in low KRN pools. Among the 56/131 (43%) informative variants, 14/56 (25%) could be validated.

Of the 51 informative KAVs in the BSLE population, 7/51 (14%) showed significant differences in allele frequency between C30 LE and C30 SE populations. To rule out the possibilities of genetic drift or stochastic sampling error, we conducted simulation to mimic the selection procedure. After simulation, one validated KAV did not pass the cutoff (FDR < 0.05) and was removed. Hence, even after accounting for drift and stochastic sampling errors, 6/51 (12%) KAVs were deemed to have been under divergent selection. We applied the simulation to the negative control variants, and they were indeed negative. Collectively, these loci account for 40% of the total between-population variance for KRN trait. Variants that are segregating in BSLE but not in GWAS populations or that were simply not detected as being KAVs in the GWAS populations may explain the remaining phenotypic variation between C30 LE and C30 SE.

### Summary of the genetic validation results

**Figure 3:**
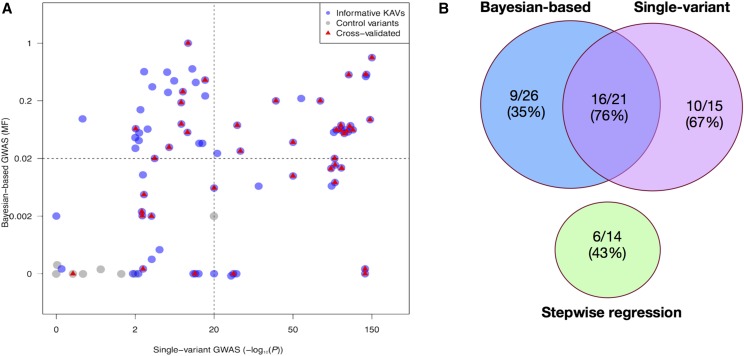
Genetic validation results of selected kernel row number associated variants (KAVs). (A) Transformed P values using single variant (SV) model and posterior model frequencies using Bayesian-based multi-variant (BMV) model were extracted and plotted for the 77 informative KAVs identified by at least one of the three GWAS models. KAVs detected only by the SV model are plotted in the lower right quadrant, KAVs detected only by the stepwise regression model are plotted as non-gray dots in the lower left quadrant, the KAVs detected only by the BMV model are plotted in the upper left quadrant, KAVs detected by both the SV and BMV models are plotted in the upper right quadrant and control variants are plotted as gray dots in the lower left quadrant. Validated KAVs are marked in red. A control variant is a genomic variant that is randomly chosen from a set of SNPs that were not associated with KRN in the initial GWAS. (B) Venn diagram of the validation results.

In summary, 40/77 (52%) of informative KAVs exhibited associations with the KRN trait in at least one of the genetic validation populations (File S5). The genetic validation results from the three GWAS models are illustrated in Figure 3. Considering all KAVs detected by each approach, the validation rates were 61% (20/33) for the single-variant approach, 43% (6/14) for the stepwise regression approach and 45% (14/31) for the Bayesian-based approach (Figure 3B). Validation rates were 67% (10/15) for KAVs detected only by the SV model, 43% (6/14) for those detected only by STR model, 35% (9/26) for KAVs detected only by the BMV model, 73% (16/22) for KAVs detected by both SV and BMV models, and 11% (1/9) for control variants that are randomly chosen from a set of SNPs that were not associated with KRN in the initial GWAS (Figure 3A). Although both the STR and BMV models had lower validation rates than the SV model, these results demonstrate that each of the three models identified validated KAVs that were not identified by other approaches. Thus, the three GWAS models are complementary.

We also obtained genotyping data for 34 KAVs reported in an earlier GWAS (Brown *et al.* 2011). Using the statistical analyses described above, 26% (9/34) of these KAVs could be validated in at least one of the three unrelated populations.

The amount of genetic recombination per Mb in maize varies substantially across the genome (Fu *et al.* 2002). To investigate whether the probability of genetic validation varies based on the amount of recombination per Mb, the 111 tested KAVs (77 from this study and 34 from Brown *et al.* (2011)) were projected onto the NAM genetic map (Buckler *et al.* 2009) using our previously published method (Liu *et al.* 2009). Recombination rates (cM/Mb) were estimated for every 10 cM window. KAVs were classified as being located in regions recombinationally “cold” (< 1 cM/Mb) or “hot” (> 1 cM/Mb) chromosomal regions. KAVs located in recombinational cold zones were 3.5 × more likely to be genetically validated than those in recombinational hot zones (Chi-square *P* value < 0.03).

## Discussion

GWAS is typically associated with high rates of false discovery (Visscher *et al.* 2012). In human studies, a second cohort is often used to genetically validate the most significant SNPs discovered in the first cohort, thereby cost effectively reducing the number of false discoveries (Sladek *et al.* 2007). To our knowledge, no large sets of genetic variants identified via GWAS as being associated with a trait of interest has been subjected to this type of genetic validation in maize. Here, we report on such a set of genetic variants that were consistently detected as being associated with KRN in both the initial GWAS and the validation populations, each of which therefore has the potential to enhance our understanding of ear development in maize.

Each GWAS model has its own strengths and weaknesses. Results from our study indicate that the three GWAS models complement each other through initial association studies and cross-validation of KAVs. In this study, genetic validation strategies that exploit the extensive genetic resources of maize were used to estimate maximum rates of cross-validation. This was accomplished by testing whether KAVs identified via the GWAS also exhibit associations with the KRN trait in independent populations. We tested not only KAVs in or near genes that had previously been associated with the KRN trait via functional analyses, but also KAVs that were not located in or near genes with prior evidence of affecting the KRN trait. There are multiple reasons for a KAV that would not be genetically cross-validated. These include biological differences in the genetic control of the KRN trait among populations and Type II errors in the validation analyses.

Overall, at least 52% (40/77) of KAVs were cross-validated in at least one of three unrelated populations. Because KRN is mainly controlled by additive effect loci (Brown *et al.* 2011; Lu *et al.* 2010), traits controlled by different modes of inheritance may yield different validation results. Because the four GWAS populations were all derived from 27 founder lines, they differ from a diversity panel with respect to the genetic base. While diversity panels exploit LD from a broader genetic base, multi-parent mapping populations are usually derived from few founders (narrow genetic base), which could also contribute to different validation results. Therefore, it may not be possible to generalize our results from these structured multi-parent mapping populations to other types of populations such as diversity panels. Further, GWAS conducted in plants often have access to immortalized genotypes and replicated observations, which provides the opportunity to better control for stochastic factors, such as environmental effects, that could affect the rate of false discovery as compared to GWAS conducted on humans or some other species.

Although the SV model had a somewhat higher genetic validation rate than the other two models (possibly at least partly because of analytic similarities between the SV model and the genetic validation experiments), each GWAS model identified validated KAVs that were not detected by the others. By definition, this means that the three GWAS models are complementary. Therefore, the use of multiple approaches or the development of a statistical model that combines their advantages, promises to enhance the power of GWAS.

The genetic validation rate of KAVs identified in this experiment (40/77 = 52%) is higher than KAVs identified in an earlier KRN GWAS study (9/34 = 26%) (Brown *et al.* 2011). The improved power of our study (which made use of data from Brown *et al.* (2011), as well as additional data generated as part of the current study) could be due to the use of three complementary GWAS models for identifying KAVs, the inclusion of more genotypes, more phenotypic data and/or higher marker density.

KAVs located in chromosomal regions with low rates of recombination (cM/Mb) were 3.5 times more likely to be genetically validated than those in chromosomal regions with high rates of recombination per physical distance. This is probably a consequence of the relationship between recombination and LD (Kim *et al.* 2007). Specifically, a KAV that is not causative but that is only linked to the causative variant is more likely to exhibit an association with the KRN trait in an independent population if it is located in a large LD block as compared to a KAV that is in a region with low LD, as a consequence of higher rates of recombination separating a marker from the functional polymorphism across different genetic backgrounds.

In conclusion, this study identified hundreds of KAVs that in combination explained 64% of phenotypic variation for KRN in lines that sample ∼60% of the genetic diversity of maize (Liu *et al.* 2003). Over 50% of KAVs that were tested could be genetically validated. In-depth analyses of KAV-linked genes will enable us to better understand the molecular and developmental processes that control variation in the KRN trait and may eventually be useful in breaking the negative correlation between KRN and ear length, thereby increasing grain yields.
